# The Hippocampus Is Coupled with the Default Network during Memory Retrieval but Not during Memory Encoding

**DOI:** 10.1371/journal.pone.0017463

**Published:** 2011-04-11

**Authors:** Willem Huijbers, Cyriel M. A. Pennartz, Roberto Cabeza, Sander M. Daselaar

**Affiliations:** 1 Swammerdam Institute for Life Sciences, Faculty of Science, University of Amsterdam, Amsterdam, The Netherlands; 2 Center for Cognitive Neuroscience, Duke University, Durham, North Carolina, United States of America; 3 Department for Psychology and Neuroscience, Duke University, Durham, North Carolina, United States of America; Université Pierre et Marie Curie, France

## Abstract

The brain's default mode network (DMN) is activated during internally-oriented tasks and shows strong coherence in spontaneous rest activity. Despite a surge of recent interest, the functional role of the DMN remains poorly understood. Interestingly, the DMN activates during retrieval of past events but deactivates during encoding of novel events into memory. One hypothesis is that these opposing effects reflect a difference between attentional orienting towards internal events, such as retrieved memories, vs. external events, such as to-be-encoded stimuli. Another hypothesis is that hippocampal regions are coupled with the DMN during retrieval but decoupled from the DMN during encoding. The present fMRI study investigated these two hypotheses by combining a resting-state coherence analysis with a task that measured the encoding and retrieval of both internally-generated and externally-presented events. Results revealed that the main DMN regions were activated during retrieval but deactivated during encoding. Counter to the internal orienting hypothesis, this pattern was not modulated by whether memory events were internal or external. Consistent with the hippocampal coupling hypothesis, the hippocampus behaved like other DMN regions during retrieval but not during encoding. Taken together, our findings clarify the relationship between the DMN and the neural correlates of memory retrieval and encoding.

## Introduction

Neuroimaging studies have identified a network of brain regions, including ventral parietal, posterior cingulate, medial frontal, and hippocampal regions, which have been consistently linked to conscious rest. These regions show more activity during passive baseline than active task conditions [1], [2], [3] and also show strong coherence during rest [4], [5], [6]. According to an influential theory, the *default mode* hypothesis, these regions form a default mode network (DMN) engaged in specific processes that normally occur during the conscious resting state [7]. This hypothesis further holds that DMN regions are continuously active, but momentarily shut down when available resources are needed for efficient cognitive performance, giving rise to deactivation in these areas. The interest in the functional significance of the DMN has increased by indications of deviations from normal DMN activity in various clinical populations, including patients with Alzheimer's dementia [8], [9], [10], [11], schizophrenia [12], [13], and autism [14]. Yet, despite all this interest, there is still considerable debate about the specific cognitive processes that are mediated by the DMN.

Interestingly, regions seemingly overlapping with the DMN have also been associated with retrieval of past events – or episodic memory retrieval. Event-related fMRI studies of episodic retrieval found that these regions show *greater activity* when previously studied items are correctly retrieved than when they are forgotten [15], [16], [17], [18], [19]. It has been suggested that these regions are involved in processes supporting successful retrieval [18]. In sharp contrast, fMRI studies focusing on the study phase of episodic memory – also referred to as memory encoding – suggest that the same regions are associated with unsuccessful encoding [20]. These studies found that these regions show less activity during encoding for items that are later remembered than for those that are forgotten [19], [21], [22], [23], [24]. We recently confirmed that these opposing patterns, of encoding decreases and retrieval increases, actually occur in overlapping brain regions [25]. Yet, the functional significance of this *encoding/retrieval flip* pattern, in relation to the DMN remains unclear.

According to one account – the *internal orienting hypothesis* – the DMN is activated during a variety of conditions involving internally-oriented attention [18], [26]. The DMN is not only active during rest and retrieval, but also during other internally-oriented task conditions, including thinking about the past and the future [27], [28], self-referential processing [29], and visual imagery [30]. At the same time, the DMN shows deactivation during demanding tasks requiring externally-oriented, rather than internal, attention [3]. Extrapolating these findings to memory, the *internal orienting* account holds that during successful retrieval, the DMN shows enhanced activity due to the orienting of attention to internalized mnemonic representations. In contrast, during successful encoding of study items, which is strongly dependent on externally-oriented attention [31], the DMN should show deactivation due to the efficient suppression of internally-oriented thoughts [3]. Despite the plausibility of this account in relation to memory, the role of internal attention in memory-related DMN activations has never been tested.

Another possibility is that the involvement of the DMN in encoding and retrieval is not related to whether attention is oriented to internal or external events but to whether the HF is coupled or uncoupled with other components of the DMN. Although resting state coherence studies have associated the HF with DMN [4], [6], [8], in memory studies HF and DMN may show similar or different activation patterns depending on the memory phase, encoding or retrieval. During successful retrieval, HF activity tends to increase just like other components of the DMN [15], [32]. During successful encoding, however, HF activity tends to increase [15], [33] whereas DMN activity tends to decrease [19], [21], [22], [23], [24]. Thus, one possible explanation of the memory functions of the DMN is that the DMN is beneficial to memory when it is coupled with the HF, as in the case of retrieval, but it is not beneficial to memory when it is uncoupled with the HF, as in the case of encoding. Although this *hippocampal coupling* hypothesis is consistent with available evidence, it has never been directly tested within the same participants and the same experiment.

**Figure 1:**
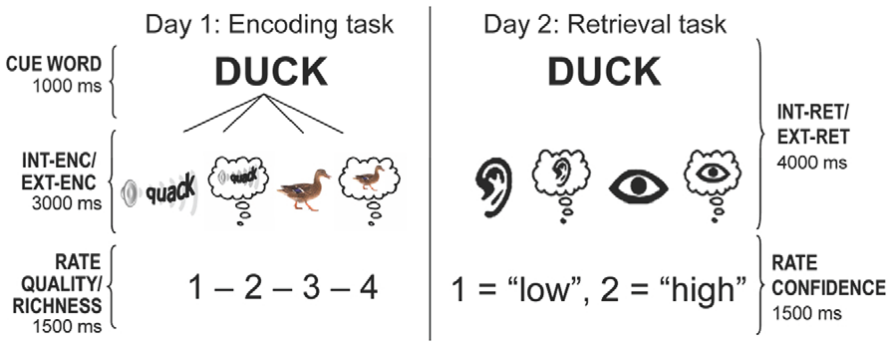
Experimental Task. Encoding trials consisted of three periods: a 1-second cue period, a 3-second encoding period, and a 1.5-second rating period. During the cue period, participants were introduced with a cue word together with an icon that indicated the trial condition. During the encoding phase, dependent on the icon, they either imagined an image or sound associated with the word (internally oriented conditions: Int-Enc) or they either perceived a sound or image associated with the word (externally oriented conditions: Ext-Enc). During the rating period, participants rated the imagery quality or perceptual richness of their experience. Retrieval trials, presented on the subsequent day, consisted of two periods, a 4-sec retrieval period, and a 1.5-sec confidence rating period. During the retrieval period, participants viewed the cue words from the previous day and retrieved the correct encoding source (1 = imagined sound, 2 = heard sound, 3 = imagined image, 4 = observed image). During the confidence rating period, they rated their confidence about their retrieval decision (“unsure”/“sure”).

In the present study, we combined resting-state and task-based fMRI to assess the *internal orienting* and *hippocampal coupling* hypotheses regarding DMN involvement in episodic encoding and retrieval. To ensure regional overlap between the DMN and episodic memory activations, we first identified the DMN based on a resting-state coherence analysis in one group of participants. Next, we probed the identified DMN regions for their involvement in episodic encoding and retrieval in a different group of participants. To test the *internal orienting* and *hippocampal coupling* hypotheses, we used a memory task including encoding and retrieval of both internally-generated (internal condition—Int) and externally-presented (external condition—Ext) events (Figure 1). During Int-Enc, subjects imagined sounds or pictures associated with a cue word (e.g., “duck”). During Ext-Enc, they listened to sounds (e.g., the “quack” sound of a duck) or observed pictures (e.g., picture of a duck) associated with the cue word. The next day, participants' retrieval of the events was tested unexpectedly with a source memory task. This paradigm allows the comparison of successful memory activations for internal and external events during both encoding and retrieval phases, and thereby, for a direct test of the *internal orienting* and *hippocampal coupling* accounts.

We tested three straightforward predictions based on previous fMRI studies of resting state coherence, encoding, and retrieval. First, on the basis of evidence from separate studies of encoding [15], [16], [17], [18] and retrieval [21], [22], [23] as well as our previous findings [19], [25], we predicted that DMN activity will be decreased during external encoding (Ext-Enc) but increased during external retrieval (Ext-Ret), that is the *encoding/retrieval flip pattern*. Second, as shown in Table 1, we tested the prediction of the *internal orienting hypothesis* that successful encoding should be associated with reduced DMN activity only when the information to be encoded is external, and hence disrupted by an internal orientation, but not when this information is internally-generated. The internal-external manipulation should not affect DMN involvement during retrieval because retrieval is always internally-oriented. Finally, we tested the prediction of the *hippocampal coupling account* that DMN activity should be associated with successful memory operations when it is coupled with the HF, which should occur during Int-Ret and Ext-Ret, but not when it is uncoupled with the HF, which should occur during both Int-Enc and Ext-Enc (Table 1).

**Table 1:** Predictions of the two accounts regarding DMN activity during successful encoding and retrieval.

	Internal orienting	Hippocampal coupling
*Successful Encoding*		
Internal	+	−
External	−	−
*Successful Retrieval*		
Internal	+	+
External	+	+

## Results

### Behavioral results

During encoding, response times for the ratings following the encoding period for Ext-Ret were 545+/−19 msec for hits and 554+/−16 msec for misses. Response times for Int-Ret were 595+/−30 msec for hits and 594+/−26 msec for misses. Response times were significantly faster for Ext-Ret than Int-Ret for both hits and misses (hits, p = 0.020; misses, p = 0.044).

During retrieval, response times for the source retrieval judgments for Ext-Ret were 1978+/−55 msec for hits and 2349+/−71 msec for misses. Response times for Int-Ret were 2142+/−65 msec for hits and 2352+/−73 msec for misses. Response times were significantly faster for Ext-Ret than Int-Ret hits but not misses (hits, p<0.0001; misses p = 0.91). Response times for the confidence ratings for Ext-Ret were 704+/−50 msec for hits and 818+/−44 msec for misses. Response times for Int-Ret were 791+/−48 msec for hits and 821+/−45 msec for misses. Once more, response times were significantly faster for Ext-Ret than Int-Ret hits but not misses (hits, p<0.0001; misses p = 0.68).

Trial percentages for Ext-Ret were 46.9%+/−2.7 hits, and 41.5%+/−2.3 misses. Trial percentages for Int-Ret were 33.8%+/−2.5 hits, and 45.6%+/−2.2 misses. In general, memory performance was better for Ext-Ret than for Int-Ret (hits, p = 0.0002; misses p = 0.10). Thus, both RT and accuracy analyses indicate that encoding and retrieval conditions were more difficult for internal than external conditions.

As behavioral evaluation of the paradigm, we expected that better imagery during encoding (Int-Enc) would result in better subsequent memory for internal events during Int-Ret. To test this prediction, we correlated the 4-point imagery quality rating with the percentage of hits during Int-Ret for each individual subject (average percentages: rating “1” = 21.23±3; “2” = 29.86±3; “3” = 44.81±3; “4” = 55.24±3). Confirming the validity of our paradigm, the average correlation was very high (R = 0.85±0.06), and very significant (p≪.0001). Thus, these results clearly indicate that high-quality imagery during Int-Enc leads to stronger memories during Int-Ret.

### fMRI results

#### Resting state coherence map

**Figure 2:**
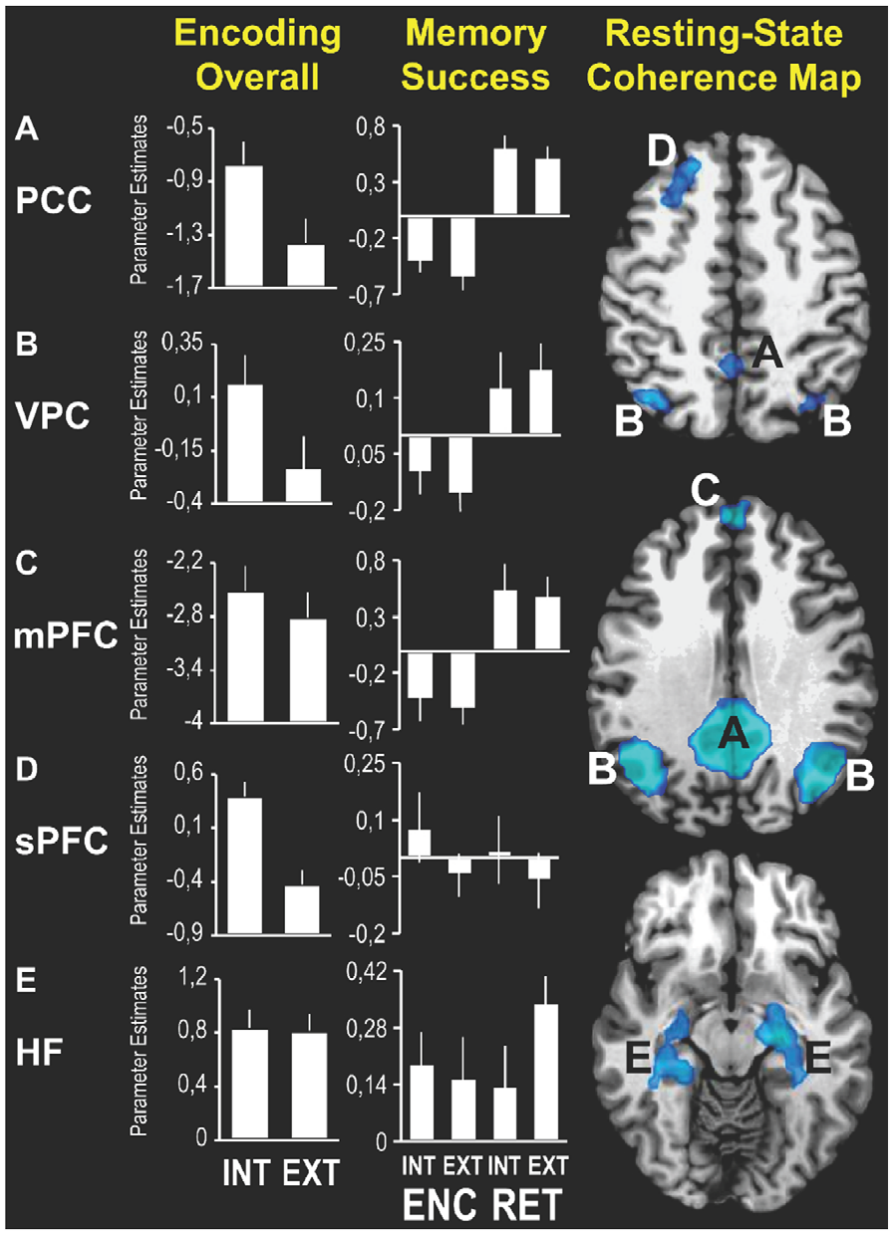
fMRI Results. Brain regions identified by the resting state analysis are depicted in blue (p<0.005, FDR corrected, cluster size >25 voxels): (A) posterior cingulate cortex, (B) ventral parietal cortex, (C) medial and (D) superior prefrontal cortex and (E) hippocampal formation. Bar graphs indicate mean cluster activity based on the memory-task (averaged over left and right sides in the case of bilateral activations). Left bar graphs show activity during encoding for internal (Int) and external (Ext) orienting conditions combined over hits and misses. Right bar graphs reflect the difference in activity between hits and misses for encoding (Int-Enc / Ext-Enc) and retrieval (Int-Ret / Ext-Ret). Vertical lines indicate SEMs.

The ICA analysis yielded only one component that showed strong regional similarities with previously reported DMN maps [4], [5], [6]. As shown in Figure 2 and Table 2, these regions included posterior cingulate cortex (23/31), ventral parietal cortex (BA 39/40), medial prefrontal cortex (BA 9/10), and left superior prefrontal cortex (sPFC). Results are reported using Montreal Neurological Institute (MNI) coordinates. Importantly, similar to previous coherence studies [4], [6], HF was also included in this component.

**Table 2:** Regions identified in resting-state analysis.

Region	Hemisphere	BA	X	Y	Z
Posterior Cingulate Ctx.	Right	7/23/31	6	−54	24
Ventral Parietal Ctx.	Left	19/39/40	−45	−75	30
	Right	19/39/40	51	−66	30
Hippocampal Formation	Left	-	−21	−39	−15
	Right	-	27	−21	−15
Medial Prefrontal Ctx.	Right	9/10	3	51	24
Superior Prefontal Ctx.	Left	8	−30	21	51

#### fMRI evaluation of the internal/external encoding paradigm

To evaluate the fMRI paradigm, we assessed whether, similar to a recent study of mental imagery by Maguire and colleagues [30], the DMN regions would show more activity during the imagined events (Int-Enc) than during the externally-presented events (Ext-Enc). As shown in Figure 2 and Table 3, a memory (successful/unsuccessful)×orientation (internal/external) repeated measures ANOVA indicated that the DMN regions, except for HF and mPFC, all showed a significant main effect of orientation, reflecting higher overall activity for Int-Enc than Ext-Enc. Together with the previous findings by Maguire and colleagues, these results clearly indicate that participants were performing the Int-Enc/Ext-Enc task as instructed.

**Table 3:** Main effects and interactions during episodic encoding.

Region	Effect	P value
Posterior Cingulate	Orientation	0.0004
	Memory	*<0.0001*
	Memory×Orientation	0.30
Ventral Parietal Ctx.	Orientation	0.0006
	Memory	*0.010*
	Memory×Orientation	0.30
medial PFC	Orientation	0.17
	Memory	*0.0024*
	Memory×Orientation	0.62
Superior PFC	Orientation	*<0.0001*
	Memory	0.78
	Memory×Orientation	0.20
Hippocampal formation	Orientation	0.19
	Memory	*0.024*
	Memory×Orientation	0.72

#### The encoding/retrieval flip pattern

Following our previous findings [19], [25], we predicted that the DMN would show the encoding/retrieval flip pattern for externally presented stimuli (Ext-Enc/Ext-Ret). Confirming this pattern, we found that the main DMN regions [26] – PCC, VPC, and mPFC – all showed greater activity during successful Ext-Ret, but less activity during successful Ext-Enc (Figure 2A–C, Tables 3 and 4). Given that our previous findings focused on PCC and VPC, the current findings not only confirm the *encoding/retrieval flip* pattern in these regions, but also extend it to mPFC.

**Table 4:** Main effects and interactions during episodic retrieval.

Region	Effect	P value
Posterior Cingulate	Orientation	0.55
	Memory	*<0.0001*
	Memory×Orientation	0.41
Ventral Parietal Ctx.	Orientation	0.48
	Memory	*0.020*
	Memory×Orientation	0.65
medial PFC	Orientation	0.95
	Memory	*0.002*
	Memory×Orientation	0.82
Superior PFC	Orientation	0.97
	Memory	0.66
	Memory×Orientation	0.53
Hippocampal formation	Orientation	0.94
	Memory	*<0.0001*
	Memory×Orientation	0.082

#### Evidence against the internal orienting account

The *internal orienting hypothesis* predicts that the internal/external manipulation should not affect DMN involvement during retrieval because retrieval is always internally-oriented. In other words, we expected increased DMN activity to be associated with successful retrieval regardless of internal or external memory orientation. As shown in Table 4 and Figure 2, results generally confirmed this prediction. Except for superior PFC, all DMN regions including HF showed a significant main effect of memory, and no memory×orientation interaction. The *internal orienting hypothesis* also predicts that successful encoding should be associated with reduced DMN activity only when the information to be encoded is external (Ext-Enc), and hence disrupted by an internal orientation, but not when this information is internal. In other words, this account predicted a memory (successful, unsuccessful)×orientation (internal, external) interaction during encoding. As shown in Figure 2 and Tables 3 and 4, none of the DMN regions showed a significant interaction. Thus, overall, our findings do not support the *internal orienting* hypothesis. As illustrated in Tables 3 and 4, whereas our retrieval findings are in agreement with the *internal orienting* hypothesis, our encoding findings clearly are not.

#### Evidence for the hippocampal coupling account

The *hippocampal coupling* account predicts that HF regions will show increased activity, together with the DMN regions during successful retrieval regardless of internal/external orientation. As noted, in line with this prediction (see Table 1), all DMN regions including HF showed a significant main effect of memory, and no memory×orientation interaction (Table 4, Figure 2). The *hippocampal coupling* account also predicts that HF regions will show increased activity during successful encoding, but the DMN regions decreased activity, regardless of internal/external orientation. In line with this prediction, as illustrated in Table 3 and Figure 2, we found that the DMN regions, except for superior PFC, showed a negative main effect of memory, whereas HF showed a positive main effect of memory. None of these regions showed a significant interaction effect between memory and orientation during encoding. Overall, these findings provide clear support for the *hippocampal coupling*, but not for the *internal orienting*, hypothesis.

### Follow-up analyses

#### Angular gyrus vs. Supramarginal gyrus/TPJ

**Figure 3:**
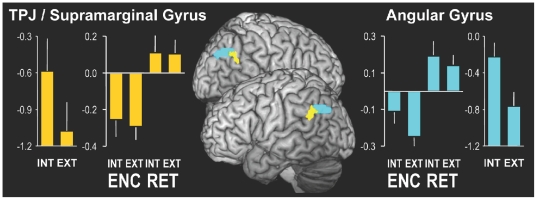
fMRI Results. Direct comparison of two ventral parietal regions: the angular gyrus (ANG – blue) and supramarginal gyrus/temporoparietal junction (TPJ - yellow). Bar graphs indicate mean cluster activity based on the memory-task (averaged over left and right sides. Left bar graphs show activity during encoding for internal (Int) and external (Ext) orienting conditions combined over hits and misses. Right bar graphs reflect the difference in activity between hits and misses for encoding (Int-Enc / Ext-Enc) and retrieval (Int-Ret / Ext-Ret). Vertical lines indicate SEMs.

The ventral parietal cortex includes two subregions: angular gyrus (ANG) and supramarginal gyrus/temporoparietal junction (TPJ). It has recently been proposed that the ventral parietal region showing a negative encoding success involves the TPJ rather than ANG [20]. At the same time, it has been proposed that ANG but not TPJ is associated with the DMN and the retrieval success network [26], [34], [35]. Although we clearly found evidence for an *encoding/retrieval flip* in overall VPC activity, it is possible that any functional differences between ANG and TPJ were obscured, since we used mean VPC cluster activity. To explore this issue further, we split the VPC region identified by the resting state scans into its two subregions by using the Wake Forest PickAtlas toolbox (http://fmri.wfubmc.edu/downloads/WFU_PickAtlas_User_Manual.pdf) and selecting “supramarginal gyrus” and “angular gyrus” (Figure 3). Next, we conducted a Region (ANG, TPJ)×Memory (Successful, Unsuccessful)×Orientation (Int/Ext) repeated measures ANOVAs for encoding and retrieval separately. As shown in Figure 3 and Table 5, besides a main effect of Region, we did not find significant interaction effects with Region during encoding. During retrieval, there was only a trending Region×Memory×Orientation interaction. In general, these results do not support a functional dissociation within VPC regarding the *encoding/retrieval flip* pattern.

**Table 5:** Angular gyrus vs. Supramarginal gyrus/TPJ.

Phase	Effect	P value
Encoding	Region	*0.029*
	Memory	*0.0002*
	Orientation	*<0.0001*
	Region×Orientation	0.87
	Region×Memory	0.10
	Memory×Orientation	0.25
	Region×Memory×Orientation	0.18
Retrieval	Region	
	Memory	*0.0084*
	Orientation	0.30
	Region×Orientation	0.89
	Region×Memory	0.23
	Memory×Orientation	0.76
	Region×Memory×Orientation	*0.082*

## Discussion

The study yielded three main findings. First, in line with our predictions, the main DMN regions, posterior cingulate cortex (PCC), ventral parietal cortex (VPC), and medial prefrontal cortex mPFC all showed an encoding/retrieval flip for externally-presented events. Decreased activity in these regions was associated with successful Ext-Enc, but increased activity with successful Ext-Ret. Second, in disagreement with the *internal orienting* account, the encoding decrease occurred regardless of whether events were internally-generated (Int-Enc) or externally-presented (Ext-Enc). Finally, in line with the *hippocampal coupling* hypothesis, the hippocampal formation (HF) showed an exception to this pattern. Increased activity was associated with successful Int-Enc and Ext-Enc as well as Int-Ret and Ext-Ret. Below, we discuss these findings in relation to the *encoding/retrieval flip* within the DMN, as well as the *internal orienting* and *hippocampal coupling* accounts of the flip pattern.

### The encoding/retrieval flip pattern

The main DMN regions, posterior cingulate, ventral parietal cortex, and mPFC, showed opposite levels of activity for successful Ext-Enc and successful Ext-Ret, i.e. the *encoding/retrieval flip* pattern. The finding that these regions show less activity during successful encoding, but increased activity during successful retrieval, confirms our previous findings [21], [25]. Based on the finding that the DMN regions tend to show deactivation during efficient cognitive performance [3], we have proposed that this pattern represents an efficient memory mechanism by which normal default mode processes, such as spontaneous thought [36], are suppressed to allow successful encoding [21], [25]. At the same time, the finding that these regions show more activity during successful retrieval is in line with the view that episodic retrieval constitutes a prominent part of the default mode [6], [26]. The present study provides strong support for this view by showing functional overlap between task-based retrieval success regions and the DMN as defined by a resting-state coherence analysis. Together with the encoding results, our findings support the idea that episodic retrieval is part of the default mode of the brain, whereas episodic encoding is not, and actually benefits from suppression of DMN activity.

### Evidence against the internal orienting account

The *internal orienting hypothesis* states that focusing attention internally would be detrimental to encoding when study items are externally-presented (Ext-Enc), but it would be beneficial when these events are internally-generated (Inc-Enc). In other words, this account predicts that *decreased* activity in the DMN regions should be associated with successful Ext-Enc, but *increased* activity with successful Int-Enc. As evaluation of our internal/external manipulation, we compared activity in the DMN regions regardless of memory. In line with previous results [30], we found that the DMN regions generally showed more activity during Int-Enc than Ext-Enc conditions. Yet, counter to the *internal orienting* account, we found that, except for HF, the DMN regions generally showed decreased activity during successful encoding regardless of internal/external orientation. This finding argues against the idea that the DMN regions underlie an internal attention system.

We should stress that our findings do not automatically discount the *internal orienting* hypothesis. As noted, the DMN is not only active during mental imagery, but during various other internally-oriented tasks, including self-referential processing [29], envisioning the future [27], and thinking about another person's perspective [37]. In this respect, it is conceivable that, in the current study, the DMN was not only active during goal-directed internal operations that are relevant for mental imagery and the encoding of internal events, but also during spontaneous internal processes that are intrusive and not relevant to the task at hand. In other words, the fact that more DMN activity is also detrimental to encoding of internal events might be because, activity for task-irrelevant internal processes outweighs the activity for task-relevant internal processes, leading to less rather than more overall activity during successful Int-Enc. Yet, as discussed below, there is an alternative attentional account that can provide a better description of our results.

### Evidence for the hippocampal coupling account

In line with the *hippocampal coupling* hypothesis, HF showed an exception to the DMN *encoding/retrieval flip* pattern. Although large portions of both left and right HF were identified by as part of the DMN by the resting state analysis, this region showed a different activation pattern. Rather than an encoding/retrieval flip and regardless of encoding orientation (Int-Enc/Ext-Enc), increased activity in HF was not only associated with successful retrieval, but also with successful encoding. This finding further substantiates overwhelming evidence indicating a critical role of HF in both encoding and retrieval [38], [39], [40]. This is the first study to clearly show that HF was activated together with the other DMN regions - as indentified independently on the basis of resting-state data - during retrieval, but not during encoding. These findings raise some important questions regarding the precise link between HF and the DMN.

Based on the fact that fMRI coherence studies [6], [8] have strongly linked HF to the DMN, it has been proposed that the DMN is actually a hippocampal memory network mediating episodic memory and other internally-oriented operations [6], [18], [26] including thinking about the future [27], self-referential processing [29], and mental imagery [30]. Recently, Hassabis and Maguire proposed that these operations all involve the common process of “scene construction”, defined as the process of mentally generating and maintaining a complex and coherent scene or event [41]. They further showed that conditions requiring scene construction without episodic memory content, such as mental imagery of fictitious events, also activate HF [30]. Moreover, patients with HF damage show pronounced deficits in mental imagery of complex fictitious scenes [42]. Animal studies also link HF to the DMN. These studies have not only shown strong connections between HF and components of the DMN at the neuroanatomical level [43], [44], but also at the neurofunctional level [45], [46]. Yet, despite these findings linking HF to the DMN, the current results clearly show that, at least during memory performance, the coherence between DMN and HF is not universal, but dependent on the type of memory operation, either encoding or retrieval. More generally, the current findings are in favor of the *hippocampal coupling* hypothesis and indicate that HF is not among the core DMN regions. Further research is necessary to characterize the precise conditions under which HF is coupled to the DMN and when it is not.

### Integrating attention and memory

Although the present results are more consistent with the *hippocampal coupling hypothesis* than with the *internal orienting account*, our data is not necessarily inconsistent with an attention account that does not emphasize the internal/external distinction. For example, according to the attention to memory (AtoM) model the role of parietal regions in episodic retrieval reflect the same attentional operations that these regions contribute to perceptual processing. In particular, this model proposes that activity in the ventral parietal cortex (VPC), which includes the temporo-parietal junction (TPJ), reflects the capture of bottom-up attention by incoming information, either from the senses (perception) or from long-term memory (retrieval). In other words, VPC mediates bottom-up attentional processes regardless of whether they information capturing processed is internal or external. This model could account for the current finding that VPC activity was not affected by the internal/external manipulation, either during encoding or during retrieval.

However, the AtoM model cannot account for the encoding-retrieval flip without additional assumptions. One of these additional assumptions was proposed by Cabeza (2008) who proposed that bottom-up capture during retrieval typically reflect incoming memories and it is therefore associated with retrieval success, whereas bottom-up capture during encoding—when the information to be encoded is constant—may reflect distractions and it is therefore associated with encoding failure. It has been argued that the VPC regions associated with retrieval success [47] and with encoding failure [20] are more posterior than the TPJ region associated with bottom-up attention to perceptual stimuli [48]. However in the present study both TPJ and VPC regions showed exactly the same pattern (Figure 3).

The bottom-up attention account of VPC activity can be integrated with the hippocampal coupling account of the encoding-retrieval flip: when the capture of bottom-up attention reflect the successful memory recovery VPC and hippocampal regions are coupled, but when the capture of bottom-up attention reflect distraction and encoding failure, VPC and hippocampal regions become uncoupled.

### Conclusion

The study yielded three main findings. First, in line with our predictions, the main DMN regions, posterior cingulate, ventral parietal cortex, and mPFC all showed an encoding/retrieval flip for externally presented events. Decreased activity in these regions was associated with successful Ext-Enc, but increased activity with successful Ext-Ret. This is the first study, to link the encoding/retrieval flip pattern directly to the brain's resting state network. Second, the encoding/retrieval flip in the DMN occurred regardless of whether events were internally-generated (Int-Enc+Int-Ret) or externally-presented (Ext-Enc+Ext-Ret). This finding argues against the idea that the DMN regions underlies and internal attention system. Third, HF showed an exception to the encoding/retrieval flip pattern. Hippocampal activity increased during both successful Int-Enc and Ext-Enc as well as Int-Ret and Int-Ret. This finding indicates that HF is not one of the core DMN regions, and is dissociated from the DMN when new memories are formed.

## Materials and Methods

### Resting state scans

#### Participants

Resting state scans were acquired from twenty-two participants (14 female, mean age 23) recruited from the University of Amsterdam community. All participants were in good health and right-handed. Their native language was Dutch and they were paid 25 euro for participation. Participants gave their written informed consent and the study met all criteria for approval of the ethical board of the Amsterdam Medical Center.

#### Data acquisition

Functional MRI images were collected on a Phillips Intera 3.0T using a 6-channel standard SENSE head coil and a T2* sensitive gradient echo sequence (96×96 matrix, TR 2000 ms, TE 30 ms, FA 80°, 34 slices, 2.3 mm×2.3 mm voxel size, 3-mm thick transverse slices). Additionally, a high-resolution T1-weighted structural scan (256×256 matrix, TR 12 ms, TE 5 ms, FOV 24 cm, 68 slices, 1 mm slice thickness) was collected.

For the resting state scans, two 8-minute rest blocks were collected from each participant. Each block consisted of a black screen with a white fixation cross-hair in the center. Although not reported here, during the resting state scans, heart rate and respiration were recorded using four electrocardiogram electrodes fixed to the subjects' chest and a respiration band placed at the level of the abdomen. Participants were instructed to keep focused on the cross-hair during scanning.

#### Analysis

Statistical Parametric Mapping (SPM5; (http://www.fil.ion.ucl.ac.uk/spm) software was used to preprocess and analyze the MR data. The images were slice-time and motion-corrected, and then normalized. First, individual normalization parameters were obtained by normalizing the segmented structural scan of each subject using the Montreal Neurological Institute (MNI) T1 template image. These normalization parameters were then applied to the functional images. Next, the normalized functional images were resliced to a resolution of 3×3×3 mm and spatially smoothed using an 8-mm isotropic Gaussian kernel.

For the resting state coherence analysis, we used the Group ICA fMRI toolbox (GIFT v2.1, http://software.incf.org/software/group-ica-toolbox-gift-and-eegift/home) developed by Calhoun and colleagues [49]. We entered subjects and the two rest runs as experimental factors and used all the default settings of the GIFT software (20 components, Infomax algorithm). Next, we averaged the two runs for each subject, and subsequently used SPM5 to conduct a random effects analysis on the resulting coherence maps. Regions were classified as significant when they exceeded an FDR-corrected threshold of p<0.005, and a cluster extent threshold of 25 voxels.

### Task-based experiment

#### Participants

Twenty-one additional participants (16 female, mean age 22) from the University of Amsterdam took part in the task-based experiment, and were paid 65 euro for participation.

#### Stimuli

The stimuli consisted of 468 Dutch cue-words (nouns) matched with 468 corresponding images and 468 corresponding sounds. The matched sounds were 2-channel stereo with a sample rate of 22 kHz, 16 bit sample size, WAV-format, with a duration of 3 seconds. The matched images consisted of 640×480 pixels, 16-bit color, BMP-format and were also presented for a duration of 3 seconds. To mimic the dynamical characteristic of sounds the images faded-in (1 sec), stayed on the screen (1 sec) and then faded-out (1 sec). The cue-words were selected so that they could call to mind both an image and a sound. For example, the word “airplane” can evoke both a visual image of a plane or the sound of its engines.

#### Procedure

The fMRI experiment consisted of an encoding phase, and a retrieval phase the next day. The encoding task consisted of six experimental runs, each containing 76 trials, yielding a total of 456 trials. Each trial consisted of three parts, a 1-second cue phase, a 3-second encoding period, and a 1.5-sec rating period (Figure 1). During the cue phase, a word was shown in the middle of the screen together with one of four icons, which indicated the specific trial condition. During the 3-second encoding period, subjects either imagined an image or sound (Internal encoding orientation – Int-Enc) associated with the cue word, or perceived an image or sound (Internal encoding orientation – Ext-Enc) associated with the cue word. During the rating period, participants rated on a 4-point scale (1 = low, 4 = high) either the subjective quality of the imagery experience during Int-Enc trials or the perceptual richness of the stimulus during Ext-Enc trials. For the purpose of the present study, these ratings were not used in the fMRI analyses. Individual encoding trials were jittered between 100 and 2100 msecs.

Before starting the actual experiment, subjects were given a brief 20-trial practice session to test the volume of the headphones and to habituate to the scanner noise. To ensure a balanced design, the cue words were randomly assigned to the conditions for each individual subject. Moreover, to ensure a sufficient number of trials in each of the four conditions, we used 132 trials for the auditory imagery trials because of expected difficulty, and 108 trials for the other conditions. The order of presentation of the stimuli was pseudorandomized in such a way that no two consecutive trials were of the same condition. Participants were instructed to keep their eyes open during imagery trials to match Ext-Enc and were not told about the memory retrieval task the next day.

The next day, participant received a surprise source retrieval task. This retrieval task also consisted of six experimental runs and included the same number of trials as the encoding task. Trial duration and jittering of the trials was also identical to the encoding task. Retrieval trials consisted of two phases. During the first phase (3000 msec), one of the cue-words from the encoding task was presented again, and participant's indicated the correct encoding source (1 = imagined sound, 2 = heard sound, 3 = imagined image, 4 = observed image). During the second phase (1500 ms), the participants rated the confidence of their judgment (“unsure”/“sure”).

#### Data acquisition

The scanner and scan parameters were the same as for the resting state scans. Auditory stimuli were presented via a MR-compatible headphone with passive noise dampening (MR Confon). Behavioral responses were collected by an MR-compatible four-button box (Lumitouch).

#### Analysis

Preprocessing of the imaging data was done in the same way as for the resting state scans. For the analysis we used SPM5 (http://www.fil.ion.ucl.ac.uk/spm/software/spm5/). Trial-related activity was assessed by convolving a vector of the onset times of the stimuli with a synthetic hemodynamic response function (HRF). The general linear model (GLM), as implemented in SPM5, was used to model the effects of interest as well as other confounding effects (scanner drift and motion). Statistical Parametrical Maps were identified for each participant by applying linear contrasts to the parameter estimates (beta weight) applying to the events of interest, resulting in a t-statistic for every voxel. Random effects analyses were employed to calculate group effects.

Separate GLMs were set up for encoding and retrieval. For each GLM, we coded four relevant trial types based on the outcome at retrieval. For encoding, these included Ext-Enc hits (later remembered) and misses (later forgotten), and Int-Enc hits and misses. Similarly for retrieval, these included Ext-Ret hits and misses, and Int-Ret hits and misses. Hits only included trials that were coupled with high confidence (“SURE”), whereas misses were collapsed across high and low confidence ratings. Low confidence hits and time outs were also included in the models, but not further analyzed.

To investigate the role of the DMN regions in encoding and retrieval we used a region-of-interest (ROI) approach using the clusters identified by the resting state analysis. Next, we conducted a performance (successful vs. unsuccessful) by orientation (internal vs. external) repeated measures ANOVAs for encoding and retrieval separately.
